# Ultrasound-Guided Perineural Dextrose Injection for Superior Cluneal Nerve Neuropathy Treatment

**DOI:** 10.7759/cureus.70229

**Published:** 2024-09-26

**Authors:** Emine Yıldırım Uslu

**Affiliations:** 1 Physical Medicine and Rehabilitation, Elazığ Fethi Sekin City Hospital, Elazığ, TUR

**Keywords:** dextrose prolotherapy, perineural dextrose injection, steroid injection, superior cluneal nerve, ultrasound-guided injection

## Abstract

Introduction: Superior cluneal nerve (SCS) entrapment neuropathy is an often overlooked cause of low back pain. Nerve block is usually applied to the sensitive point on the iliac crest where Tinel positivity is detected, with a mixture of a corticosteroid and a local anesthetic. Dextrose injection is one of the treatment methods. There are not enough studies comparing the effectiveness of dextrose and steroid injections. In this study, it was planned to compare these two methods.

Materials and methods: This retrospective study included 16 patients who underwent ultrasound-guided steroid injection and 17 patients who underwent ultrasound-guided dextrose injection with the diagnosis of SCS neuropathy at Elazığ Fethi Sekin City Hospital Polyclinics between 01.09.2023 and 28.03.2024. Patients were evaluated with visual analog scale (VAS) and Roland-Morris Disability Questionnaire (RMDQ) scores before and after the procedure at week 1

Results: While VAS and RMDQ scores were similar in both groups before the procedure, VAS and RMDQ scores at week 1 were significantly lower in the dextrose group.

Conclusion: Perineural dextrose injection appears to be a safe and effective alternative to steroid injections in the treatment of SCS neuropathy.

## Introduction

Low back pain is a musculoskeletal problem that is common in society and is known to be mostly caused by nonspecific causes and causes disability [1]. Lumbar disc herniation, spinal stenosis, spondylolysis, spondylolisthesis, facet joint arthropathy, and sacroiliac joint dysfunction are frequently considered in the differential diagnosis. Superior cluneal nerve (SCS) entrapment neuropathy is an often overlooked cause of low back pain. It is thought to be responsible for the pain in 12-14% of all patients with low back pain [2,3].

The SCS is a purely sensory nerve that originates from the lower thoracic and lumbar posterior nerves and provides cutaneous innervation to the lower lumbar and hip. It can cause back and leg pain by being trapped where it penetrates the thoracolumbar fascia near the iliac crest [4]. In the clinic of SCS entrapment neuropathy, in addition to back pain, leg pain that often mimics radiculopathy is also observed [5]. Intermittent claudication may be observed. Prolonged standing, sitting, flexion, extension, and contralateral rotation increase the pain [2]. The most important examination finding is the detection of Tinel-like signs on the posterior iliac crest. The occurrence of numbness and pain in the area of ​​nerve distribution due to compression of the trigger point in this region and the disappearance of pain with nerve block are diagnostic [2].

Postural exercises, SCS sliding exercises, and modifications in daily life activities are recommended for treatment; however, nerve block is often applied to the sensitive point on the iliac crest where Tinel positivity is detected, with a mixture of a corticosteroid and a local anesthetic [6]. Dextrose injection is also one of the treatment methods [7]. There are no sufficient studies comparing the effectiveness of dextrose and steroid injections. In this study, it was planned to compare these two methods.

## Materials and methods

This study was conducted retrospectively in accordance with the principles of the Declaration of Helsinki after receiving approval from the Ethics Committee of the Faculty of Medicine of Fırat University (date: 11.03.2024/number: 22958). Informed voluntary consent forms were obtained from the participants. The study included 16 patients who underwent ultrasound-guided steroid injection and 17 patients who underwent ultrasound-guided dextrose injection with the diagnosis of SCS neuropathy between 01.09.2023 and 28.03.2024 at the Elazığ Fethi Sekin City Hospital Polyclinics.

Patients with mechanical low back pain radiating to the iliac crest and hip for more than three months, tenderness observed on palpation in the posterior iliac crest, and numbness or increased pain complaints in the nerve distribution area on palpation were included in the diagnostic injection test with the preliminary diagnosis of SCS neuropathy. 5 cc of procaine was injected by targeting the apex of the posterior iliac crest with the in-plane technique under US guidance. The test was considered positive in patients whose pain was relieved by 50% or more within 10 minutes after the injection. Patients with a positive diagnostic injection test, aged between 18 and 60 years, and pain unresponsive to oral medical treatments were included in the study. Nine patients with concomitant lumbar radiculopathy, malignancy, chronic disease, or history of inflammatory disease were excluded from the study.

All patients selected for injection treatment were applied in the prone position. A pillow was placed under the abdomen to reduce lumbar lordosis. The skin was sterilized with an antiseptic solution. The tissues around the iliac crest were visualized with a linear US probe. A 21-gauge 40 mm needle was advanced to the top of the iliac crest using an in-plane technique. In the steroid injection group, 2.63 mg betamethasone sodium phosphate + 5 mg betamethasone dipropionate was injected, and in the dextrose group, 5 cc 5% dextrose was injected. All patients were recommended SCS shift exercises, posture, and pelvic tilt exercises after the injection.

Results before and one week after the procedure were evaluated using visual analog scale (VAS) [8] and Roland-Morris Disability Questionnaire (RMDQ) [9] scores.

Data were analyzed using IBM SPSS Statistics for Windows, Version 22 (Released 2013; IBM Corp., Armonk, New York, United States). According to the characteristics of the variables, percentage, mean±standard deviation, and median were given. Wilcoxon, student t-test and Pearson correlation tests were used. Statistical significance was accepted as p<0.05.

## Results

Age, body mass index (BMI), and gender distribution of both groups were evaluated. No statistically significant difference was observed between the two groups (Table 1).

**Table 1:** Demographic data of dextrose and steroid groups BMI: Body Mass Index p<0.05 was considered significant

Parameter	Dextrose Group (n=17)	Steroid Group (n=16)	p
Gender distribution Female (%)	47%	53%	0.723
Age (years) (mean±SD)	46.05±5.26	48.13±5.39	0.280
BMI (kg/m^2^) (mean±SD)	26.62±2.42	27.61±2.39	0.257

The VAS and RMDQ scores of the group before and one week after the procedure were evaluated. While the VAS and RMDQ scores before the procedure were similar in both groups, the VAS and RMDQ scores in the first week were significantly lower in the dextrose group (Table 2).

**Table 2:** Comparison of VAS and RMDQ scores before and one week after the procedure in the dextrose and steroid groups VAS: Visual Analog Scale; RMDQ: Rolland-Morris Disability Questionnaire p<0.05 was considered significant

Parameter	Dextrose Group (n=17)	Steroid Group (n=16)	p
Preprocedure VAS (mean±SD)	5.81±0.98	6.13±0.957	0.278
1st week VAS (mean±SD)	1.63±1.08	2.50±0.516	0.016
Preprocedure RMDQ (mean±SD)	13.31±1.92	13,38±1.147	0.378
1st week RMDQ (mean±SD)	4.31±1.62	5,81±1.328	0.009

In the dextrose and steroid groups, VAS and RMDQ scores decreased significantly at the first follow-up point compared to before the procedure (Table 3).

**Table 3:** Temporal change of VAS and RMDQ scores in dextrose and steroid groups VAS: Visual Analog Scale; RMDQ: Rolland-Morris Disability Questionnaire

	Parameter	Preprocedure	1st week	p
Dextrose group (n=17)	VAS (mean±SD)	5.81±0.98	1.63±1.08	0.00
	RMDQ (mean±SD)	13.31±1.92	4.31±1.62	0.00
Steroid group (n=16)	VAS (mean±SD)	6.13±0.957	2.50±0.516	0.00
	RMDQ (mean±SD)	13,38±1.147	5,81±1.328	0.00

The recovery rates were determined by calculating the percentage change in the VAS scores of the patients before and after the procedure. The VAS recovery rate was 68.48±25.41 in the dextrose group; the VAS recovery rate was 58.87±7.38 in the steroid group. The correlation of the VAS recovery rates with age and BMI was analyzed in both groups. No correlation was observed between the recovery rates with age and BMI (Table 4).

**Table 4:** Correlation analysis of the recovery rate in VAS scores with BMI and age in the dextrose and steroid groups VAS: Visual Analog Scale; BMI: Body Mass Index p<0.05 was considered significant

Parameter	Age		BMI	
	r	p	r	p
VAS Improvement rate in the dextrose group (n=17)	-0.321	0.226	-0.332	0.209
VAS improvement rate in the steroid group (n=16)	0.152	0.573	-0.326	0.218

## Discussion

This study compared the effectiveness of percutaneous dextrose injection and steroid injection under ultrasound guidance in the treatment of SCS neuropathy. As a result of the study, it was determined that both methods provided a significant decrease in pain and disability scores, but dextrose injection was more effective than steroid injection.

Although noninvasive treatments for SCS neuropathy include topical nonsteroidal anti-inflammatory drugs or physical modalities such as transcutaneous electrical nerve stimulation, the most commonly used treatment method is nerve block, which is applied to the trigger point on the iliac crest with a mixture of a local anesthetic and a steroid [10,11]. Injections with different solutions have also found a place in the treatment of SCN neuropathy. Pure local anesthetic injections have been reported to be effective for patients with severe symptoms who want a rapid analgesic effect [12]. Mahli et al. treated four patients with SCN compression after bone graft harvest from the iliac crest using alcohol neurolysis [13]. Inklebarger et al. reported that they treated two patients with 12.5% ​​dextrose combined with phenol, glycerin, and lidocaine [14].

Steroid injection is a commonly used treatment for SCS neuropathy [10]. Steroids are known to have pain-relieving effects when applied locally by preventing tissue edema and inflammatory cell infiltration that impede the regular gliding motion of the nerve, but they also have the potential to cause nerve tissue damage and systemic side effects. The use of steroids, such as dexamethasone, has been extensively investigated as an adjuvant in various nerve blocks, including brachial plexus blocks [15]. However, potential neurotoxic effects associated with high-dose steroid administration have raised concerns about their long-term safety. In addition to the pain-relieving effects of steroids when applied locally, they are also known to have potential damage to nerve tissue and systemic side effects. Therefore, safe and effective alternative treatment methods are needed in the management of neuropathic pain.

5% dextrose has an isotonic concentration and is increasingly used in the treatment of peripheral neuropathies. It has been reported to be effective in the treatment of carpal tunnel syndrome [7,16]. It is also thought to be effective in the treatment of other peripheral neuropathies. It has been reported to be non-neutral in animal and human studies and, unlike hypertonic dextrose (>10%), it does not have a pro-inflammatory effect [17-19]. Although the mechanism of therapeutic action is not fully known, it is thought to prevent the discharge of pro-nociceptive substances substance P and calcitonin gene-related peptides by down-regulating capsaicin-sensitive receptors [20]. It is also thought to increase blood flow by separating the entrapped nerve from the adjacent soft tissue with mechanical hydrodissection effect and reduce the effects of chronic ischemic damage [21]. It is the most commonly used fluid for perineural hydrodissection because it is the most harmless fluid for the nerve tissue, surrounding vascular tissue, and soft tissues and has good anesthetic and analgesic effects [7]. There are views that neuroregenerative mechanisms that have not yet been explained may also be effective [22].

Previous studies have reported promising results with perineural dextrose injection for SCS neuropathy. Siahaan et al. conducted a case series of four patients who received 10 ml of 5% dextrose injection and observed significant decreases in VAS scores at two-week follow-up [23]. Similarly, Chang et al. reported 80% symptom improvement in patients with SCS neuropathy who were injected with a combination of 1% lidocaine and 5% dextrose [24]. Consistent with these findings, our study also showed significant early decreases in pain scores after dextrose injections. Chen et al. compared the efficacy of dextrose and steroids in ulnar neuropathy and found dextrose to be superior [25]. Similarly, Nasiri et al. compared dextrose and steroids in carpal tunnel syndrome and observed that dextrose was effective and safe in the early period [26]. The available literature comparing the efficacy of dextrose and steroids for SCS neuropathy is limited. Our results suggest that dextrose injections may be superior to steroid injections in the early management of SCS neuropathy. We thought that this result may be due to the hydrodissection effect of the higher volume dextrose injection.

Although the results of this study suggest that perineural dextrose injection may be more effective than steroid injection in the treatment of SCS neuropathy, it is important to consider the limitations of the study and the need for further research. The sample size was relatively small, and the long-term effects of these interventions were not assessed. Additionally, the safety profile of perineural dextrose injection, particularly with respect to potential neurotoxicity, requires further investigation.

## Conclusions

There are several important implications of the current study. Perineural dextrose injection appears to be a safe and effective alternative to steroid injections for the treatment of SCS neuropathy. Dextrose is a relatively inexpensive, readily available, and non-neurotoxic agent, making it an attractive option for clinicians. In addition, avoiding potential long-term steroid-related side effects is a major advantage of the dextrose approach. Higher-quality, larger-scale studies with longer follow-up periods are needed to draw more definitive conclusions about the comparative efficacy and safety of these two treatment modalities. Clinicians should carefully evaluate the available evidence and consider the potential benefits and risks for each patient when deciding on the most appropriate treatment approach for SCN neuropathy.
